# Nanoparticles increase human bronchial epithelial cell susceptibility to respiratory syncytial virus infection via nerve growth factor‐induced autophagy

**DOI:** 10.14814/phy2.13344

**Published:** 2017-07-12

**Authors:** Sreeparna Chakraborty, Vincent Castranova, Miriam K. Perez, Giovanni Piedimonte

**Affiliations:** ^1^ Department of Pediatrics West Virginia University School of Medicine Morgantown West Virginia; ^2^ Department of Pharmaceutical Science West Virginia University School of Pharmacy Morgantown West Virginia; ^3^ Pediatric Institute and Children's Hospital The Cleveland Clinic Cleveland Ohio

**Keywords:** Air pollution, asthma, bronchiolitis, lung injury, neurotrophins

## Abstract

Cytotoxic and neuroinflammatory effects of TiO_2_ nanoparticles (TiO_2_‐NP) in human airways are mediated by nerve growth factor (NGF), which is also implicated in the pathophysiology of respiratory syncytial virus (RSV) infection. We tested the hypothesis that exposure to TiO_2_‐NP results in increased susceptibility to RSV infection and exacerbation of airway inflammation via NGF‐mediated induction of autophagy in lower respiratory tract cells. Human primary bronchial epithelial cells were exposed to TiO_2_‐NP for 24 h prior to infection with recombinant red RSV (rrRSV). Expression of NGF and its TrkA and p75^NTR^ receptors was measured by real‐time PCR and fluorescence‐activated cell sorting (FACS). Autophagy was assessed by beclin‐1 expression analysis. Cell death was studied by FACS after annexin V/propidium iodide staining. rrRSV infection efficiency more than doubled in human bronchial cells pre‐exposed to TiO_2_‐NP compared to controls. NGF and its TrkA receptor were upregulated in RSV‐infected bronchial cells pre‐exposed to TiO_2_‐NP compared to controls exposed to either rrRSV or TiO_2_‐NP alone. Silencing NGF gene expression with siRNA significantly inhibited rrRSV infection. rrRSV‐infected cells pre‐exposed to TiO_2_‐NP also showed increase in necrotic cell death and reduction in apoptosis, together with 4.3‐fold increase in expression of the early autophagosomal gene beclin‐1. Pharmacological inhibition of beclin‐1 by wortmannin resulted in increased apoptotic rate along with lower viral load. This study shows that TiO_2_‐NP exposure enhances the infectivity of RSV in human bronchial epithelial cells by upregulating the NGF/TrkA axis. The mechanism of this interaction involves induction of autophagy promoting viral replication and necrotic cell death.

## Introduction

A recent report of the World Health Organization revealed that exposure to pollution is associated with more than one in four deaths among children younger than 5, and 570,000 children in this age range die every year from respiratory infections attributable to pollution. Indeed, respirable particulate matter – especially nanosized particles with aerodynamic diameter ≤0.1 *μ*m – has been linked to inception and exacerbations of asthma and chronic obstructive pulmonary disease (Thurston and Ozkaynak 1992; Bascom 1996). Owing to their smaller size and higher surface area, inhaled virus‐sized particles exhibit high deposition in the lower respiratory tract and frequently act as carriers for copollutants of industrial and biological origin (e.g., gases, chemicals, bacteria, viruses) (Oberdorster 2001). Titanium dioxide nanoparticles (TiO_2_‐NP) have drawn special attention because of their extensive use in commercial applications (Xia et al. 2006), such as paints, photocatalysts (Sun et al. 2004), antibacterial films or sprays (Shieh et al. 2006), sunscreens and cosmetics, as well as their potential for causing inflammation and injury of the lower airways in a dose‐dependent manner (Oberdorster 2001; Chen and Schuster 2006; Grassian et al. 2007).

While the individual effects of pollution and infections on the respiratory tract have been studied extensively, exposure to these agents is frequently simultaneous, resulting in potentially augmented pathological responses. Respiratory syncytial virus (RSV) is the most common cause of hospitalization in children under the age of 2 years (Wright and Piedimonte 2011). In addition, RSV adversely affects the elderly and immunocompromised individuals, causing severe lower respiratory tract infection and pneumonia (Falsey 2007). This virus first replicates in epithelial cells of the upper respiratory tract and then migrates throughout the lungs, where it induces pulmonary inflammation, epithelial cell necrosis, and mucus plugging leading to airway obstruction and lung hyperinflation (Simoes 2008; Wright and Piedimonte 2011).

Previous studies have shown that during the course of RSV infection, neuroimmune interactions driven by neurotrophic pathways play a critical role in the pathophysiology of airway inflammation (Othumpangat et al. 2009). In particular, the prototypical nerve growth factor (NGF) and its tropomyosin receptor kinase A (TrkA) high‐affinity receptor are overexpressed in RSV‐infected airways leading to persistent changes in the distribution and reactivity of sensory and motor nerves, which results in nonspecific airway hyperreactivity (Piedimonte 2003). Our previous studies have also shown that RSV interferes with apoptotic pathways of host airway epithelial cells via NGF‐mediated mechanisms (Othumpangat et al. 2009).

Mounting evidence indicates that NGF‐mediated cytoprotection involves modulation of autophagic pathways (Rosso et al. 2015; Franco et al. 2016; Wang et al. 2017). This evolutionarily conserved process of cellular self‐degradation allows the selective digestion of damaged or unneeded cytosolic substructures into amino acids and fatty acids that can be recycled for macromolecular synthesis and energy production essential for cell survival and stress adaptation. While autophagy is vital for cellular innate immunity (Morris et al. 2011), viruses like enterovirus 71, influenza A, human parvovirus B19, and dengue virus 2 are able to repurpose autophagy to enhance their replication efficiency (Nakashima et al. 2006; Huang et al. 2009; Heaton and Randall 2011). In the present study, we tested the hypothesis that previous exposure to ultrafine titanium dioxide (TiO_2_‐NP) upregulates the NGF/TrkA axis, resulting in increased susceptibility to RSV infection via induction of autophagy in lower respiratory tract cells.

## Methods

### TiO_2_‐NP

Commercial grade nanoparticles also known as Aeroxide TiO_2_ P‐25 (Evonik Degussa Corporation, Parsippany, NJ) were a kind gift from Dr. Min Ding, Pathology and Physiology Research Branch, Health Effects Laboratory Division, National Institute for Occupational Safety and Health. The endotoxin level of this P‐25 sample has been shown to be below detection levels (Dr. Min Ding, personal communication). Stock solutions of TiO_2_‐NP (10 mg/mL) were prepared in sterile distilled water by sonicating for 5 min on ice using an ultrasonic probe sonicator (Sonics & Materials, Newtown, CT). To measure size and agglomeration pattern, sonicated TiO_2_‐NP suspensions in distilled water and in serum‐free cell culture medium (10 *μ*g/mL) were tested with a particle size analyzer (Microtrac, Montgomeryville, PA) using the dynamic light‐scattering (DLS) technique.

### Cells

Human bronchial epithelial cells from multiple donors were purchased from PromoCell (Heidelberg, Germany) and were maintained in the recommended media and supplements provided by the vendor. Each experiment was repeated using cells from different donors throughout the study to control for host genetics and environment. The number of passage times for each airway section was matched in each experiment and never exceeded five passages.

### Virus

Recombinant RSV derived from the RSV‐A2 strain and expressing the gene for red fluorescent protein (rrRSV) was a kind gift from Dr. Mark E. Peeples (Columbus Children Research Institute, Columbus, OH) and Dr. Peter Collins (National Institutes of Health, Bethesda, MD). Virus stock was serially diluted and added to HEp‐2 cell monolayers in 96‐well plates for 24 h, and infected cells with internalized red virus were counted by fluorescent microscopy. rrRSV titer was determined with a modified plaque‐forming unit (PFU) assay, and it was calculated as follows: (number of infectious units) × (inverse of dilution)/(volume of inoculum). The titer of virus stock used in this study was 4.8 × 10^6^ PFU/mL.

To study the effect of TiO_2_‐NP pre‐exposure on rrRSV infection efficiency in human bronchial epithelium, cells were grown to 70–80% confluency in culture wells and treated with serum‐free medium containing 10 *μ*g/mL of sonicated nanoparticle suspension for 24 h. We have shown previously that this dose of TiO_2_‐NP is sufficient to induce NGF signaling without substantial cell necrosis (Chakraborty et al. 2017). After incubation, the spent medium was removed and cells were washed with prewarmed media and incubated with rrRSV at multiplicity of infection (MOI) of 0.5 at 37°C with 5% CO_2_ for 24 h. Following infection, cells were harvested by trypsinization, RNA was extracted, and viral copy number was measured with the PrimerDesign™ kit for human RSV. To confirm that the observed effects were caused by actively replicating virus and not by exposure to virion proteins or culture medium, selected experiments were repeated using aliquots of rrRSV irradiated with UV light for 30 min to inactivate the viral nucleic acid (Haeberle et al. 2008).

### Real‐time PCR analysis

Total RNA was isolated from TiO_2_‐NP pre‐exposed bronchial epithelial cells infected with rrRSV and nonexposed control cells using the RNeasy kit (Qiagen, Valencia, CA). This RNA was used for RT‐PCR analysis using the SYBR green one‐step RT‐PCR master mix (Qiagen) in an ABI 7500 real‐time cycler (Applied Biosystems). Validated NGF, TrkA and p75^NTR^ primers were purchased from SABiosciences (Rockville, MD). Relative expression was calculated for each sample using the ^ΔΔ^Ct method after normalization against the endogenous controls hypoxanthine phosphoribosyltransferase‐1, peptidylprolyl isomerase A (cyclophilin A), and beta‐2‐microglobulin (HPRT1, PPIA, and B2M, respectively; RealTimePrimers, Elkins Park, PA).

### Immunofluorescence

Nanoparticle pre‐exposed cells infected with rrRSV and nonexposed controls were isolated by trypsinization, fixed with 4% paraformaldehyde (PFA) in phosphate‐buffered solution (PBS) at room temperature for 10 min, and permeabilized with 0.3% Triton X. Nonspecific binding was discriminated by adding human IgG (Sigma‐Aldrich, St. Louis, MO) for 20 min. Cells were incubated with primary antibodies for NGF, its p75^NTR^ receptor (Santa Cruz Biotechnology, Santa Cruz, CA) followed by Alexa‐488‐conjugated secondary antibodies (Santa Cruz Biotechnology), and TrkA conjugated with allophycocyanin (R&D Systems, Minneapolis, MN). Cells were then analyzed using FACS Calibur with Cell Pro software (BD Biosciences, San Jose, CA). Data were further analyzed with Windows Multiple Document Interface (WinMDI, flow cytometry version 2.9, Scripps Research Institute, La Jolla, CA).

For confocal imaging studies, bronchial cells grown to 70% confluence on poly‐l‐lysine‐coated glass coverslips were treated with TiO_2_‐NP and infected with rrRSV. No monolayer disruption or cell lifting due to treatment was noted. Infected cells were rinsed in PBS, fixed with 4% PFA at room temperature for 10 min, permeabilized with 0.3% Triton X, and treated with antibodies for NGF, p75^NTR^ conjugated with Alexa‐488 (Santa Cruz Biotechnology), or TrkA conjugated with allophycocyanin (R&D Systems) as described above. Cells were mounted in Prolong Gold antifade reagent (Invitrogen, Carlsbad, CA) containing 4',6 diamidino‐2‐phenylindole (DAPI). Images were collected and analyzed using a Zeiss LSM510 confocal microscope (Carl Zeiss, Jena, Germany) with a 405‐diode laser to excite DAPI and an argon laser to excite the secondary antibodies.

### Cell death

To distinguish between necrosis and apoptosis, 10^6^ cells/mL of unfixed cells pre‐exposed to nanoparticles and infected with rrRSV along with appropriate controls were costained with 5 *μ*g/mL each of propidium iodide (PI) and fluorescein isothiocyanate (FITC)‐conjugated annexin V (BD Pharmingen). Cells counts were acquired with a FACS Calibur flow cytometer and cell fractions in each quadrant were analyzed using Cell Quest Pro software (BD Biosciences, Franklin Lakes, NJ). Cells in the lower right quadrant represented early apoptosis, while cells in the upper right quadrant represented late apoptosis. Necrosis was represented by cells in the upper left quadrant. Fluorescence compensation on the flow cytometer was adjusted to minimize overlap of the FITC and PI signals. A total of 10–20,000 events were acquired for each sample from three different experiments.

### NGF knockdown by siRNA

The role of NGF during rrRSV infection of nanoparticle pretreated bronchial cells was investigated by silencing its expression with a specific siRNA (Dharmacon, Chicago, IL) transfected with Lipofectamine™ 2000 (Invitrogen, Carlsbad, CA). Following manufacturer's instructions, the medium was replaced 6 h after transfection with serum‐free culture medium to allow the cells to recover for another 42 h. RT‐PCR was performed to verify NGF knockdown efficiency, rrRSV copy numbers, and beclin‐1 expression.

### Autophagy

To determine whether RSV infection induces autophagy in nanoparticle pretreated bronchial cells, we measured expression of beclin‐1, a key component of PI3K/beclin‐1 complex that plays an important role in the initial stage of autophagy. Gene expression was assessed by RT‐PCR with validated beclin‐1 primers (SABiosciences, Rockville, MD), while protein expression was analyzed by flow cytometry and confocal microscopy with a beclin‐1 primary antibody (Cell Signaling Technology, MA).

Autophagy in rrRSV‐infected cells was further evaluated by blocking beclin‐1 expression with the inhibitor wortmannin (Calbiochem, La Jolla, CA). Briefly, bronchial cells were grown to 70% confluency in six‐well culture dishes and treated with 50 nmol/L wortmannin for 30 min, while control cells were incubated with DMSO vehicle (Di Bartolomeo et al. 2010). After changing the medium, cells were thoroughly washed with prewarmed media and then treated with nanoparticle suspension and rrRSV. RT‐PCR measured the changes in beclin‐1 gene expression following treatment with wortmannin. rrRSV infection efficiency was assessed with the PrimerDesign™ kit. Treated cells were immunostained on coverslips as described above, and the changes in rrRSV infection efficiency were further confirmed by confocal microscopy. Cell death patterns following inhibition of autophagy were measured with a FITC annexin V apoptosis detection kit (BD Pharmingen).

### Statistical analysis

Data are expressed as mean ± standard deviation (SD). Statistical analysis was performed with the Student's *t*‐test or with ANOVA followed by Tukey–Kramer's post hoc test using SigmaStat 3.5 (Systat Software Inc., San Jose, CA) software. *P* < 0.05 was considered significant.

## Results

### Nanoparticle characterization

Aeroxide P25 TiO_2_‐NP are manufactured as small aggregates in the 100 nm range consisting of several primary particles each having a mean diameter of approximately 21 nm. However, TiO_2_‐NP sonicated to a suspension and analyzed by DLS formed larger agglomerates, exhibiting increased mean diameter in both distilled water (0.14 *μ*m) and culture media (0.62 *μ*m), with uniform dispersion.

### Effect of nanoparticle exposure on RSV infection

**Figure 1:**
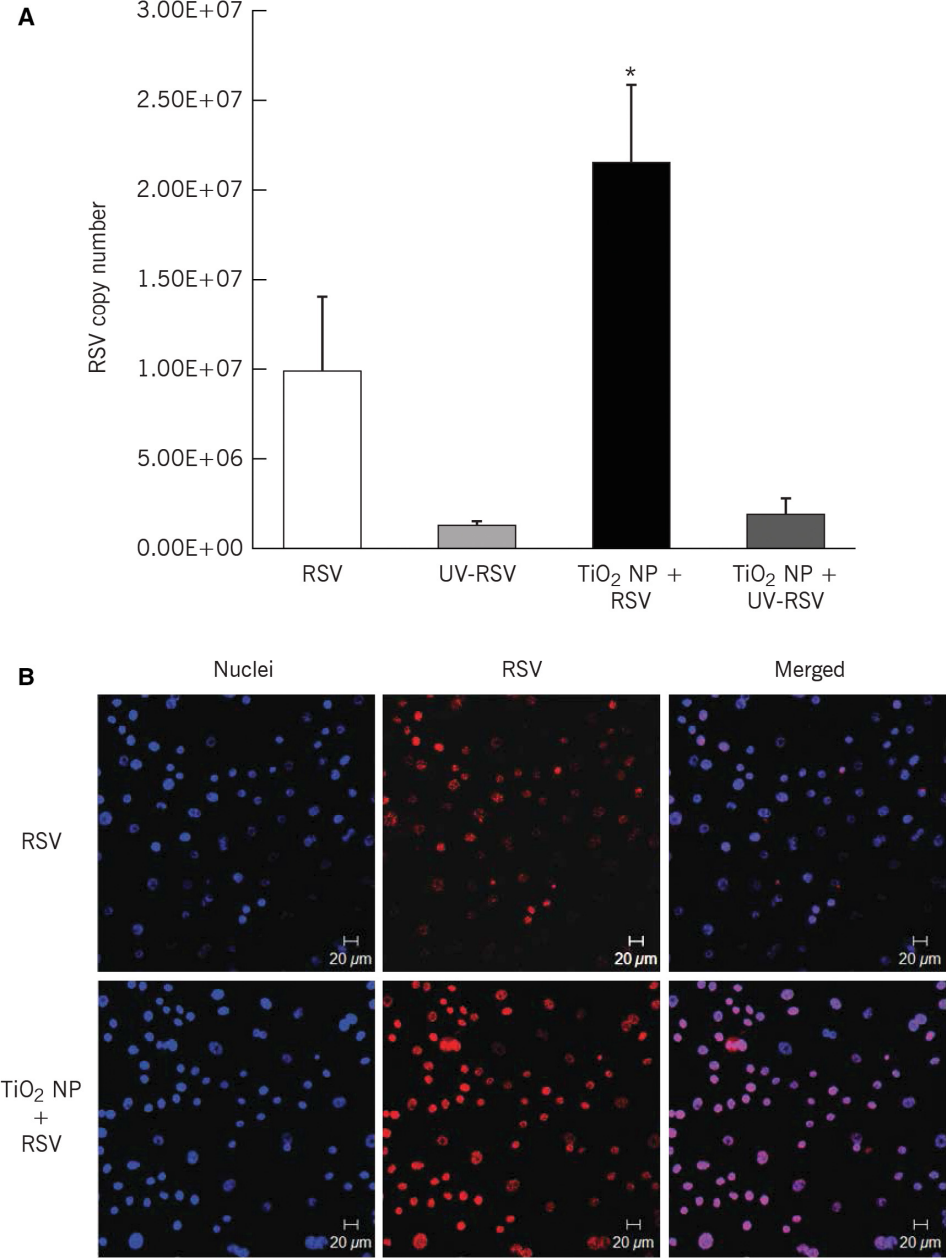
Titanium dioxide nanoparticle pre‐exposure increases susceptibility to RSV infection in bronchial cells. (A) Bronchial cells were treated with 10 *μ*g/mL of TiO_2_‐NP for 24 h and then infected with RFP‐expressing RSV (rrRSV) at 0.5 multiplicity of infection (MOI) for 24 h. Real‐time PCR analysis showed increased rrRSV copy number in nanoparticle‐treated bronchial cells. Data are expressed as mean ± SD (*n* = 5 experiments). *P < 0.05 compared to nonexposed rrRSV‐infected cells. (B) Cells grown on coverslips were treated as above and analyzed by confocal microscopy for rrRSV infection. TiO_2_‐NP pre‐exposed bronchial cells showed increased red fluorescent protein expression.

Pre‐exposure of human bronchial epithelial cells to TiO_2_‐NP prior to infection with rrRSV doubled the efficiency of viral replication (*P* < 0.05; Fig. 1A). This effect required active viral replication, as it was abolished by UV inactivation of the viral nucleic acid. Confocal microscopy confirmed higher density of cells exhibiting red fluorescence as a result of increased viral replication and expression of the RFP transgene after exposure to TiO_2_‐NP (Fig. 1B).

**Figure 2:**
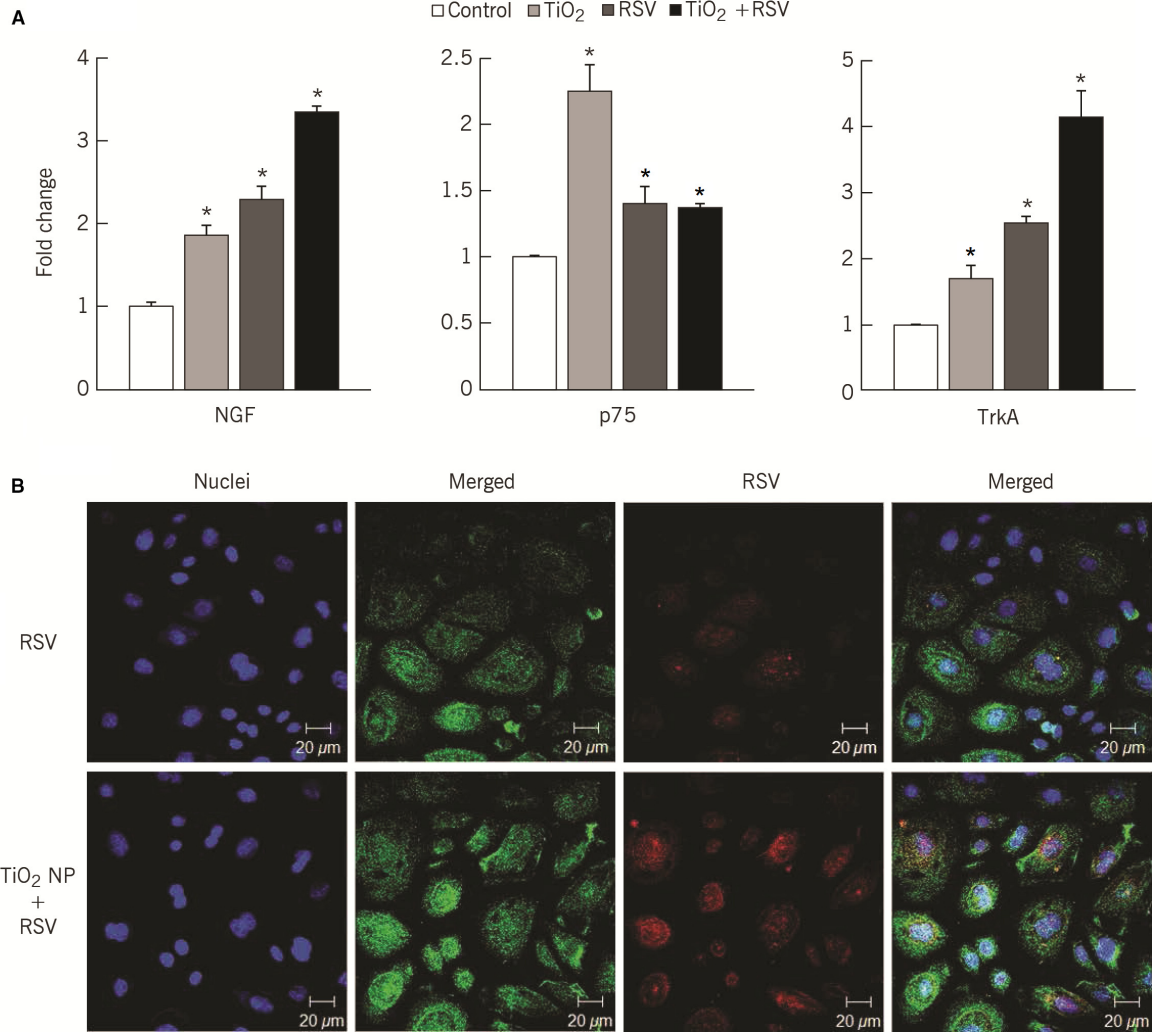
Gene expression of NGF and its p75^NTR^ and TrkA receptors in TiO_2_‐NP pre‐exposed human bronchial epithelial cells infected with RSV. (A) Real‐time PCR analysis of RNA from bronchial cells pretreated with 10 *μ*g/mL of TiO_2_‐NP and then infected with RFP‐expressing RSV (rrRSV) at 0.5 multiplicity of infection (MOI) for 24 h showed differential NGF, p75^NTR^, and TrkA expression. Data are expressed as mean ± SD (*n* = 5 experiments). *P < 0.05 compared to nonexposed controls. (B) TiO_2_‐NP pre‐exposed rrRSV‐infected bronchial cells showed increased red fluorescence concomitant with NGF upregulation. Nuclei were stained with DAPI. Images shown are representative of three independent experiments.

**Figure 3:**
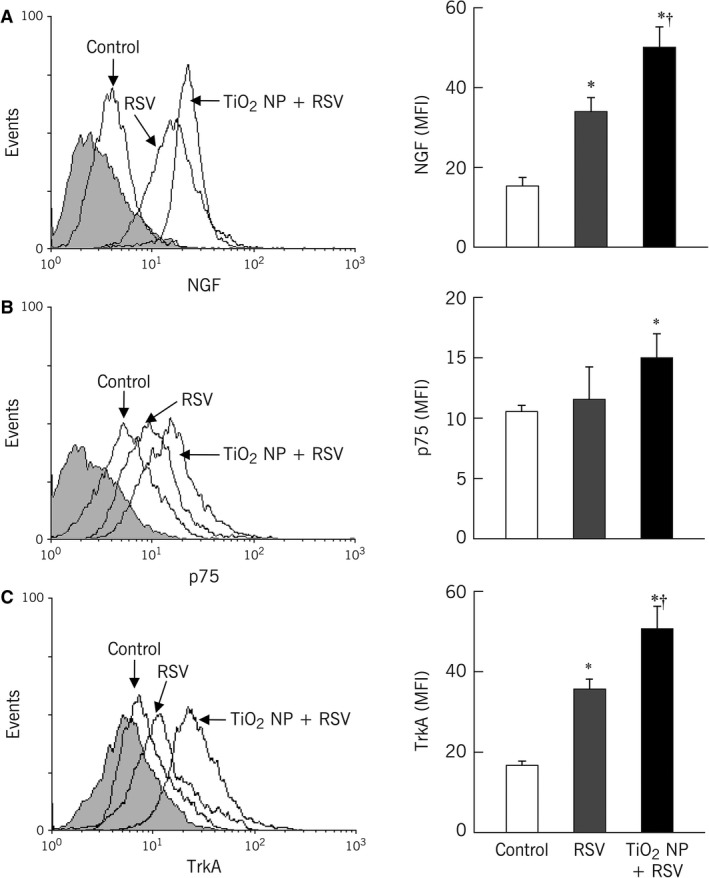
Pre‐exposure to TiO_2_‐NP alters protein expression of NGF and its receptors in bronchial cells during RSV infection. Cells grown on coverslips were pretreated with 10 mg/mL of TiO_2_‐NP for 24 h, then infected with RFP‐expressing RSV (rrRSV) at 0.5 multiplicity of infection (MOI) for 24 h. Cells were harvested at 48 h and stained with specific antibody or matched isotype control (shaded histogram). Noninfected cells notexposed to nanoparticles were used as controls. Changes in (A) nerve growth factor (NGF); (B) p75 neurotrophin receptor (p75NTR); and (C) tropomyosin receptor kinase A (TrkA) protein expression were measured by flow cytometry. Pre‐exposure to TiO_2_‐NP potentiated the increase in NGF and TrkA concentrations caused by rrRSV, whereas p75NTR expression increased only when cells were infected after exposure to TiO_2_‐NP. Geometric mean fluorescent intensity (MFI) is shown in bar graphs. Data are expressed as mean ± SD (*n* = 3 experiments). *P < 0.01 compared to control; †*P* < 0.01 compared to rrRSV‐infected cells.

TiO_2_‐NP exposure alone significantly upregulated gene expression of NGF (1.9‐fold, *P* < 0.01), p75^NTR^ (2.2‐fold, *P* < 0.001), and TrkA (1.8‐fold, *P* < 0.05; Fig. 2A). Compared to TiO_2_‐NP, rrRSV infection alone led to larger increments in NGF and TrkA gene expression (2.3‐fold and 2.5‐fold, respectively), but only minor increase in p75^NTR^ transcripts (1.5‐fold). However, when bronchial cells were exposed to TiO_2_‐NP followed by rrRSV infection, NGF and its high‐affinity receptor TrkA gene expression increased in additive fashion (*P* < 0.001), whereas p75^NTR^ receptor expression decreased significantly compared to levels measured after TiO_2_‐NP alone (*P* < 0.01). Changes in gene expression were reflected in stronger NGF protein immunoreactivity observed by confocal microscopy (Fig. 2B). In addition, changes in NGF, TrkA, and p75^NTR^ proteins were quantified by flow cytometry (Fig. 3), confirming that pre‐exposure to TiO_2_‐NP increased NGF and TrkA concentrations in rrRSV‐infected bronchial cells. In contrast, p75^NTR^ receptor protein expression did not show any change from control after rrRSV infection alone, and increased slightly only when cells were infected after being pre‐exposed to TiO_2_‐NP (Fig. 3B).

**Figure 4:**
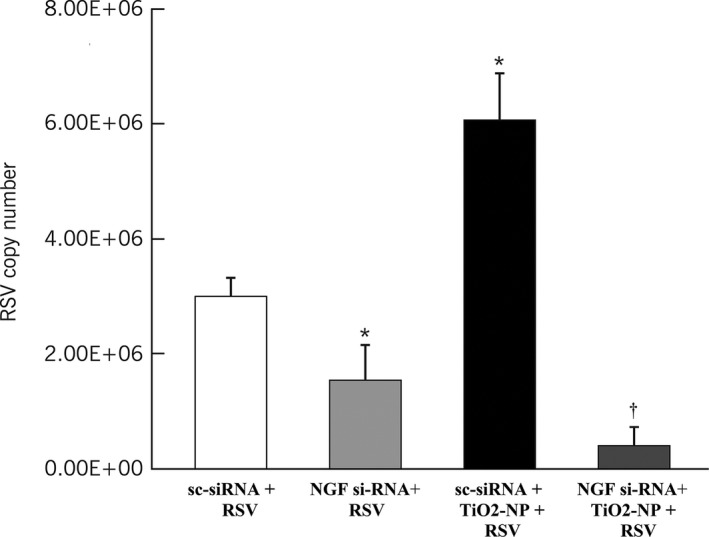
TiO_2_‐NP‐induced NGF modulates RSV infection in bronchial cells. Scrambled siRNA (sc‐siRNA) and NGFsiRNA‐transfected bronchial cells were pretreated with 10 *μ*g/mL of TiO_2_‐NP for 24 h, then infected with RFP‐expressing RSV (rrRSV) at 0.5 multiplicity of infection (MOI) for 24 h. Cells were harvested and RNA isolated for rrRSV copy number analysis by RT‐PCR. Knockdown of NGF resulted in reduced viral titer in infected bronchial cells. Data are expressed as mean ± SD (*n* = 3 experiments). *P < 0.05 compared to sc‐siRNA‐transfected rrRSV‐infected cells; ^†^ *P* < 0.001 compared to TiO_2_‐NP‐pretreated sc‐siRNA‐transfected rrRSV‐infected cells.

To investigate the role of nanoparticle‐induced NGF in modulating rrRSV infection, rrRSV‐infected bronchial cells were transfected with NGF‐specific siRNA or scrambled control siRNA (Fig. 4). Bronchial cells pre‐exposed to TiO_2_‐NP and transfected with scrambled siRNA showed significant increase in rrRSV copy number compared to nonexposed cells (2.3‐fold increase, *P* < 0.001). Knockdown of NGF gene expression by specific siRNA transfection virtually blocked rrRSV replication in bronchial cells pre‐exposed to TiO_2_‐NP (*P* < 0.002), suggesting that NGF upregulation by nanoparticles predisposes human airways to more severe subsequent infection by RSV.

**Figure 5:**
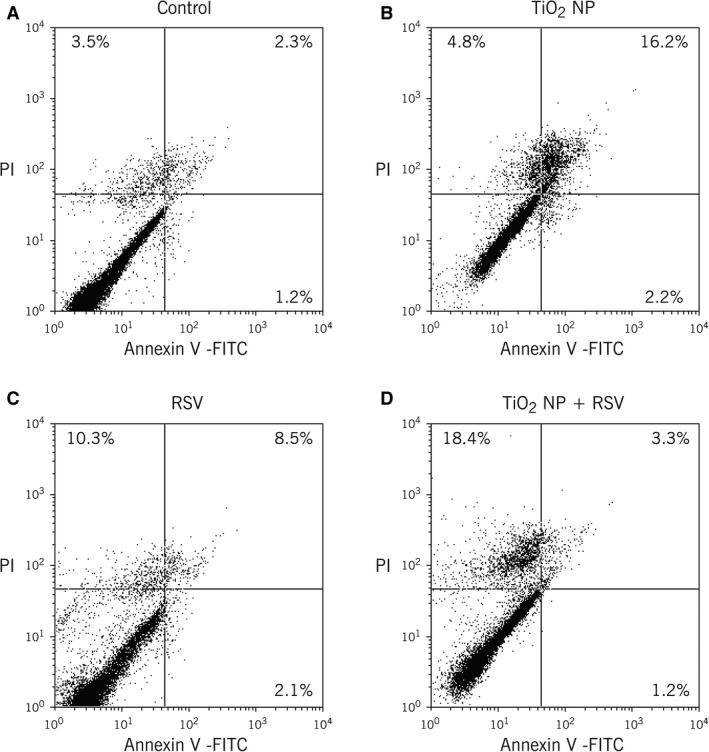
Altered cell death patterns in TiO_2_‐NP‐pretreated bronchial epithelial cells during RSV infection. Annexin V and propidium iodide staining was used for detection of apoptosis and necrosis in rrRSV‐infected TiO_2_‐NP‐pretreated bronchial epithelial cells. Data are expressed as percentage (%) of total cells. Compared to control cells (A), increased apoptosis was noted in only TiO_2_‐NP‐exposed cells (B), with apoptosis and necrosis in only rrRSV‐infected cells (C). Necrosis was markedly increased in rrRSV‐infected, TiO_2_‐NP‐pretreated bronchial cells with concomitant decrease in apoptosis (D). Data are expressed as the mean ± SD (*n* = 3 experiments).

Bronchial cells pre‐exposed to TiO_2_‐NP showed increased rate of apoptotic death as compared to untreated controls (Fig. 5A and B). In contrast, both apoptosis and necrosis increased in cells infected with rrRSV without previous exposure to TiO_2_‐NP (Fig. 5C). However, when cells were pre‐exposed to TiO_2_‐NP before infection with rrRSV a decrease in apoptosis was noted along with a rise in necrosis compared to controls (Fig. 5D), suggesting that nanoparticle pre‐exposure can alter host cells death pattern in human airways favoring subsequent RSV infection.

### Role of autophagy

**Figure 6:**
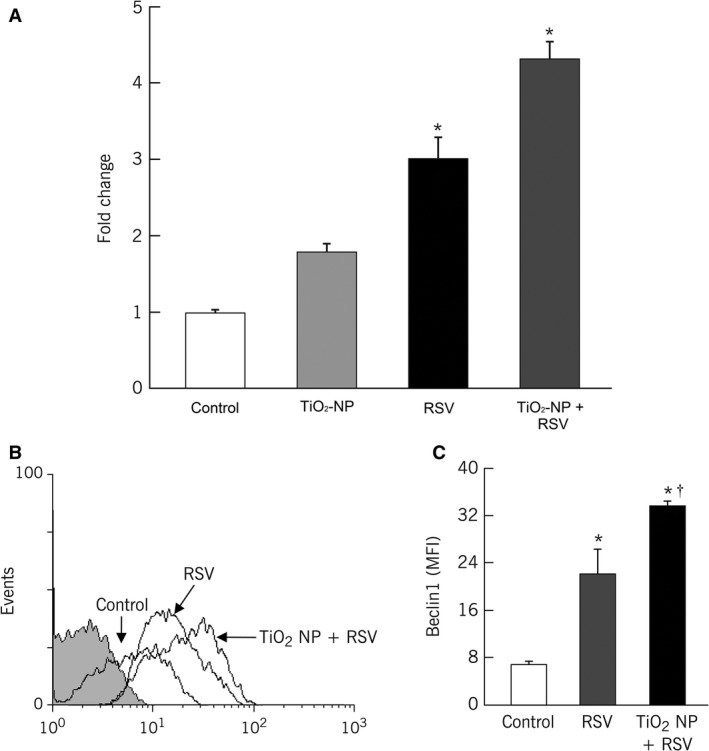
Upregulation of autophagosomal gene beclin‐1 expression in TiO_2_ preexposed RSV‐infected bronchial epithelial cells. (A) Real‐time PCR analysis of RNA from bronchial cells pretreated with 10 *μ*g/mL of TiO_2_‐NP and then infected with RFP‐expressing RSV (rrRSV) at 0.5 multiplicity of infection (MOI) for 24 h showing differential beclin‐1 mRNA expressions. Data are expressed as mean ± SD (*n* = 5 experiments). *P < 0.001 compared to controls. (B) Bronchial cells treated as above were harvested at 48 h and stained with specific antibody or its matched isotype control (shaded histogram). Noninfected cells not exposed to nanoparticles were used as controls. Changes in protein expression were measured by flow cytometry. Geometric mean fluorescent intensity (MFI) is shown in the bar graphs. Data are expressed as the mean ± SD (*n* = 3 experiments). *P < 0.001 compared to control; ^†^ *P* < 0.001 compared to only rrRSV‐infected cells.

Induction of autophagy in bronchial cells was assessed by measuring changes in beclin‐1 gene expression (Fig. 6A). TiO_2_‐NP exposure significantly upregulated beclin‐1 mRNA in bronchial cells infected with rrRSV compared to untreated controls (4.3‐fold increase, *P* < 0.001), noninfected cells exposed to TiO_2_‐NP (*P* < 0.001), and nonexposed rrRSV‐infected cells (*P* < 0.01). UV‐inactivated rrRSV failed to produce any change in beclin‐1 expression (data not shown), indicating that active viral replication is necessary to induce autophagy. Gene expression data were concordant with protein expression levels measured by flow cytometry (Fig. 6B), supporting the involvement of autophagic pathways in the stress response of bronchial cells to RSV infection.

**Figure 7:**
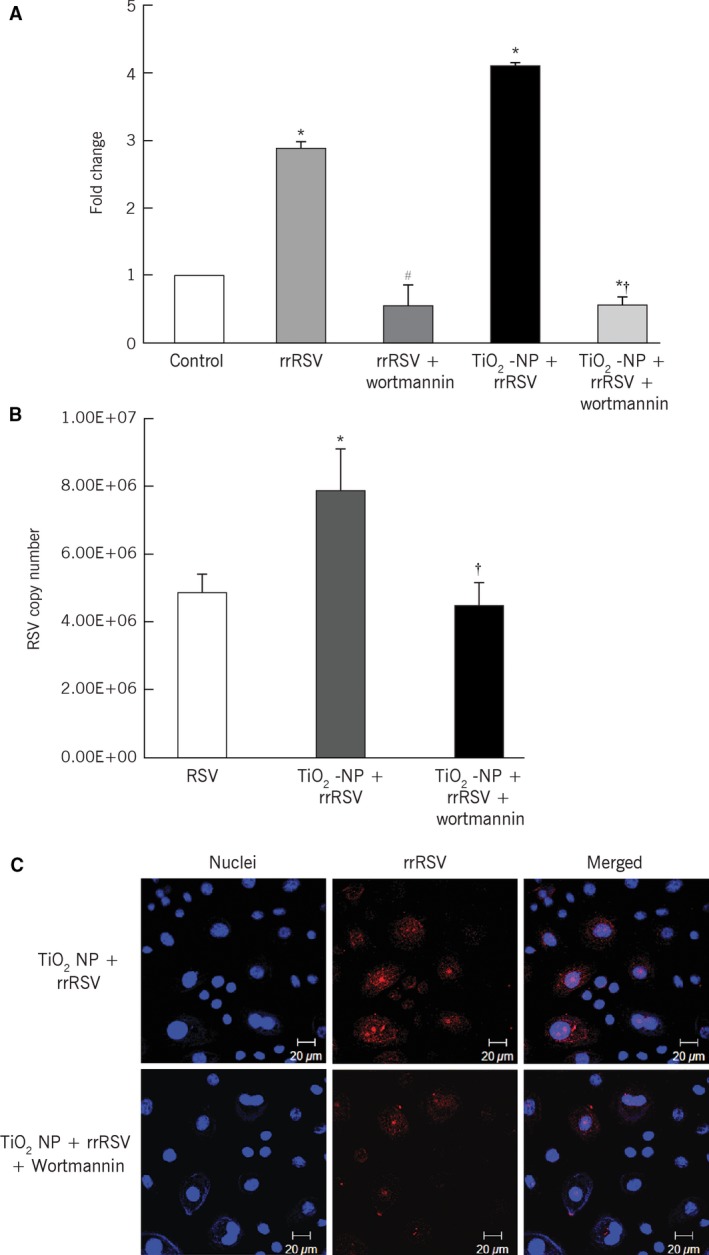
Inhibition of autophagy with the PI3‐kinase inhibitor wortmannin. (A) Bronchial cells were treated with 50 nM of wortmannin followed by 10 *μ*g/mL of TiO_2_‐NP for 24 h, and then infected with RFP‐expressing RSV (rrRSV) at 0.5 multiplicity of infection (MOI) for another 24 h. Real‐time PCR showed a decrease in beclin‐1 gene expression following wortmannin treatment. *P < 0.01 compared to control; ^#^ *P* < 0.01 compared to rrRSV alone; ^†^ *P* < 0.01 compared to rrRSV‐infected, TiO_2_‐NP‐pre‐exposed cells. (B) Decrease of viral copy numbers in rrRSV‐infected, TiO_2_‐NP‐pretreated bronchial cells following inhibition of autophagy. Data are expressed as the mean ± SD (*n* = 4 experiments). *P < 0.001 compared to rrRSV; ^†^ *P* < 0.001 compared to rrRSV‐infected, TiO_2_‐NP‐pre‐exposed cells. (C) Cells grown on coverslips and analyzed by confocal microscopy showed a decrease in red fluorescence indicating lower viral load. Data shown are representative of three independent experiments.

**Figure 8:**
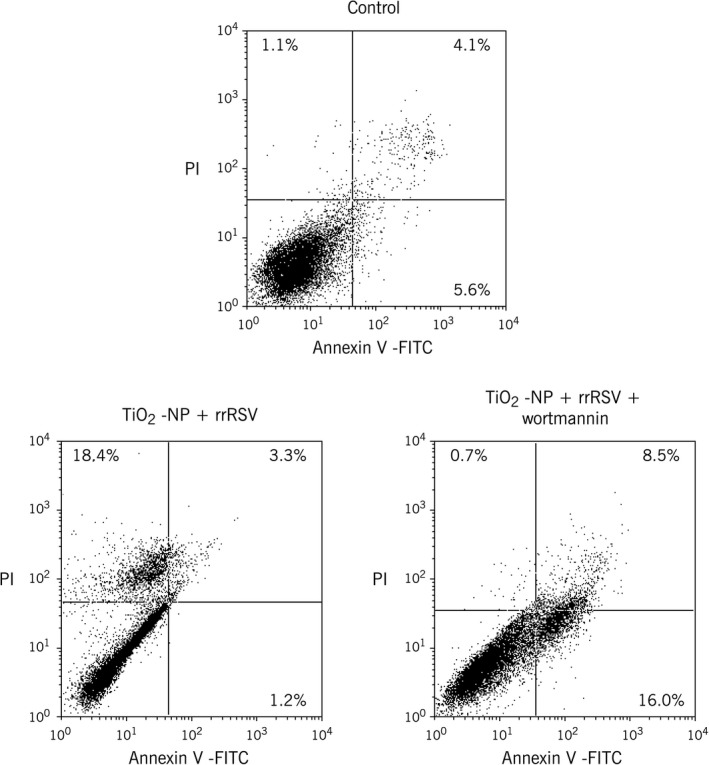
Inhibition of beclin‐1 with wortmannin increases apoptosis in TiO_2_‐NP‐exposed bronchial epithelial cells infected with RSV. Bronchial cells were pretreated with 50 nmol/L of wortmannin followed by 10 *μ*g/mL of TiO_2_‐NP for 24 h, and then infected with RFP‐expressing RSV (rrRSV) at 0.5 multiplicity of infection (MOI) for another 24 h. Annexin V and propidium iodide staining showed increased apoptosis in cells following autophagy inhibition. Data shown are representative of three independent experiments and are expressed as percentage (%) of total cells.

To further confirm the role of autophagy in RSV infection, beclin‐1 was selectively inhibited with wortmannin. Following treatment with wortmannin, significant downregulation of beclin‐1 mRNA expression was noted in rrRSV‐infected bronchial cells either with or without pre‐exposure to TiO_2_‐NP compared to respective controls without wortmannin (*P* < 0.01; Fig. 7A). Furthermore, inhibition of autophagy lowered the efficiency of virus replication (*P* < 0.001; Fig. 7B). Confocal imaging studies confirmed that blocking beclin‐1 activity decreases rrRSV infection in bronchial cells (Fig. 7C), indicating that autophagy promotes virus replication in the human respiratory tract. Finally, blocking autophagy decreased necrosis and increased apoptosis in rrRSV‐infected bronchial cells pre‐exposed to TiO_2_‐NP (*P* < 0.01; Fig. 8).

## Discussion

The fundamental process of nanotechnology is the synthesis of engineered virus‐sized particles, whose unique properties offer great opportunities for the development of revolutionary industrial and biomedical applications. The explosion of their worldwide distribution is exponentially multiplying the likelihood of environmental and human exposure. Yet, our understanding of the health and safety aspects (i.e., biocompatibility) of nanomaterials is quite limited and highly controversial. In this study, we explored for the first time interactive effects between TiO_2_‐NP, a high‐production nanoparticle prototypic in size to ultrafine ambient air particles, and the most common agent of lower respiratory infections in early childhood, human RSV. Nanosized titanium dioxide particles were used in this study primarily because of our interest in respirable particle size, their ability to deposit in the distal respiratory tract and evade phagocytosis, and lack of preexisting data on the interactive responses between RSV and respirable particulates in human. Since the lower airways are the natural target of RSV infection, we carried our experiments on human bronchial epithelial cells (Othumpangat et al. 2009).

Our study shows for the first time that exposure of human bronchial epithelial cells to nanosized particles prior to infection with RSV increases significantly the efficiency of viral replication, and this effect requires upregulation of the NGF/TrkA axis. Specifically, TiO_2_‐NP pretreatment followed by RSV infection had additive effect on NGF synthesis along with concomitant upregulation of its high‐affinity receptor TrkA and lower expression of the low‐affinity p75^NTR^ receptor. In previous work, we had demonstrated that this same pattern of neurotrophins expression prevents the apoptotic death of RSV‐infected airway epithelial cells, thereby enhancing epithelial viability with a mechanism similar to that previously described in macrophages infected with human immunodeficiency virus (HIV) and essential for the host cell to survive while supporting the production of new viral particles (Garaci et al. 1999).

Thus, present data confirm that intracellular bioavailability of NGF is a critical determinant of the efficiency of RSV replication. Indeed, silencing endogenous NGF gene expression with a specific siRNA before nanoparticulate exposure dramatically decreased RSV copy number. On the other hand, increased NGF expression combined with augmented expression of the p75^NTR^ receptor in noninfected cells exposed to nanoparticles signaled a proapoptotic response, which is consistent with our previous studies where TiO_2_‐NP exposure led to increased apoptosis via JNK pathway in human nasal, tracheal, bronchial, and alveolar cells (Chakraborty et al. 2017). The model emerging from combining the available experimental evidence is that differential expression of NGF and its cognate receptors serves as a survival mechanism allowing infected cells to tolerate the stress of viral invasion, but at the same time can turn into a critical virulence factor permitting successful replication of viral genes and ultimately leading to necrotic lysis of the host cells with spreading of the infection.

To further confirm the role of ultrafine particulate exposure on cell viability during RSV infection, bronchial cells were analyzed after staining with annexin V and propidium iodide. Noninfected bronchial cells exposed to nanoparticles showed higher percentage of apoptosis, which is consistent with the increased expression of p75^NTR^ receptor gene measured by RT‐PCR. Nonexposed RSV‐infected cells showed an increase in necrotic cell population when compared to noninfected cells, consistent with the pathologic findings of airway epithelial necrosis and accumulation of inflammatory cells in RSV‐positive bronchiolitis (Wright and Piedimonte 2011). Finally, RSV‐infected bronchial cells pre‐exposed to nanoparticles showed a greater increase in the percentage of necrotic cells, with approximately one fifth of cells staining positive for propidium iodide by 24 h, but also maintained a lower rate of apoptosis by upregulating NGF‐TrkA signaling (Othumpangat et al. 2009). Similarly, RSV infection of HEp‐2 cells induces the antiapoptotic factor IEX‐1L (Domachowske et al. 2000), and inhibition of phosphatidylinositol‐3‐kinase (PI3K) results in more rapid apoptosis via the NF‐*κ*B pathway (Thomas et al. 2002).

In light of the mounting evidence that NGF‐mediated cytoprotection is mediated through autophagic mechanisms (Rosso et al. 2015; Franco et al. 2016; Wang et al. 2017), we also investigated whether this cellular self‐degradation process limits apoptosis during RSV infection by measuring expression of the early marker beclin‐1. This mammalian homolog of yeast Atg6/Vps30 is at the heart of a regulatory complex for the class III PI3K/hVps34, and its activity is essential during autophagosome formation (Liang et al. 1999; Yue et al. 2003). Our data show significant upregulation of beclin‐1 expression in RSV‐infected bronchial cells pre‐exposed to respirable‐size nanoparticles. However, nanoparticle exposure alone did not affect bronchial cells autophagy, suggesting a critical role of RSV in modulating the PI3K/beclin‐1 complex. Recent studies have also shown activation of autophagy pathways in several other viral infections, like influenza A virus, human immunodeficiency virus 1, hepatitis C virus, poliovirus, and coxsackievirus B3, supporting the importance of this evolutionarily conserved mechanism of stress adaptation in modulating viral replication efficiency by preserving the homeostasis of host cells (Wong et al. 2008; Zhou et al. 2009; Kim et al. 2010).

The role of autophagy during nanoparticulate exposure and subsequent RSV infection of distal airway epithelial cells was further elucidated by blocking the autophagic flux with the selective beclin‐1 inhibitor wortmannin, which interferes with PI3K activity by competitively occupying its ATP‐binding site (Yuan and Cantley 2008). The present study shows that inhibition of PI3K/beclin‐1 activity leads to significant decrease of RSV replication in bronchial cells, highlighting the importance of early autophagy in infected human airways. Furthermore, inhibiting beclin‐1 activity significantly increased apoptosis in RSV‐infected bronchial cells pre‐exposed to TiO_2_‐NP. Similar studies have shown that beclin‐1 inhibition can also expose latent apoptotic pathways in immortalized human hepatocytes infected with hepatitis C virus (Shrivastava et al. 2011).

Results of the present study indicate that pretreatment of human bronchial epithelial cells with TiO_2_‐NP (10 *μ*g/mL) 24 h prior to infection with RSV at a relatively low MOI increased infection efficiency by twofold. At first light, this result may seem unexpected, since metal nanoparticles, such as titanium dioxide, cadmium oxide, zinc oxide, or silver, are commercially employed as antimicrobial agents (Sun et al. 2008; Rezaei‐Zarchi et al. 2010). However, this antimicrobial activity is expressed upon direct exposure of bacteria or virus to nanoparticles, while in our study host epithelial cells, not virus, were exposed to TiO_2_‐NP. In contrast to our study with TiO_2_‐NP, nanocarbon black did not alter cell titers of herpes virus after a 2‐h primary infection (Sattler et al. 2017). However, nanocarbon black and double‐walled carbon nanotubes did increase viral titers in alveolar macrophages or mouse lungs with a latent rather than ongoing infection (Sattler et al. 2017). Pulmonary exposure of mice to TiO_2_‐NP 5 days prior to respiratory RSV infection increased the severity of RSV‐induced pneumonia, although lung viral titers were not significantly affected, perhaps due to the high level of viral inoculation used (Hashiguchi et al. 2015). Furthermore, Shvedova et al. (2008) reported that pulmonary exposure to single‐walled carbon nanotubes increased respiratory infectivity of *Listeria monocytogenes* bacteria, decreased bacterial phagocytosis by macrophages, and depressed the production of the antimicrobial agent nitric oxide by macrophages. Further investigation is needed to determine the potency of various nanoparticles in enhancing susceptibility to infection and to elucidate mechanisms by which this enhanced infectivity is expressed.

In conclusion, our data suggest that exposure of the lower airway epithelium to nanosized environmental particles makes the respiratory tract more susceptible to subsequent RSV infection. This effect is mediated by upregulation of the NGF/TrkA axis with concurrent amplification of autophagic pathways. Autophagy allows infected cells made susceptible by prior exposure to ultrafine particles to better adapt to the stress of viral invasion, and prevents apoptotic cell death while the virus completes its replication cycle that will ultimately lead to necrotic cell lysis. Based on these data, we stress the importance of monitoring hidden biological risks potentially associated with the rapid diffusion of novel nanomaterials. We also speculate that pharmacological manipulation of apoptotic and autophagic pathways may increase the resistance of human airways against airborne biological, physical, and chemical agents.

## Conflict of Interest

None declared.
